# Correlation Between Soluble Endothelial Adhesion Molecules and Nitric Oxide Metabolites in Sickle Cell Disease

**DOI:** 10.3390/medsci7010001

**Published:** 2018-12-20

**Authors:** Charles Antwi-Boasiako, John Ahenkorah, Eric S. Donkor, Bartholomew Dzudzor, Gifty B. Dankwah, Kate H. Otu, Robert Aryee, Charles Hayfron-Benjamin, Andrew D. Campbell

**Affiliations:** 1 Department of Physiology, School of Biomedical and Allied Health Sciences, University of Ghana, Accra +233, Ghana; gdankwah@gmail.com (G.B.D.); Bobby200055@gmail.com (R.A.); chayfron-benjamin@ug.edu.gh (C.H.-B.); 2 Department of Anatomy, School of Biomedical and Allied Health Sciences, University of Ghana, Accra +233, Ghana; jahenkorah@ug.edu.gh; 3 Department of Medical Microbiology, School of Biomedical and Allied Health Sciences, University of Ghana, Accra +233, Ghana; ericsdon@hotmail.com; 4 Department of Medical Biochemistry, School of Biomedical and Allied Health Sciences, University of Ghana, Accra +233, Ghana; bdzudzor@ug.edu.gh; 5 Department of Nursing and Midwifery, Greenhills School of Health Sciences, Accra +233, Ghana; kateboasiako@yahoo.co.uk; 6 Department of Anaesthesia, School of Medicine and Dentistry, University of Ghana, Accra +233, Ghana; 7 Center for Cancer and Blood Disorders Children’s National Medical Center George, Washington University School of Medicine and Health Sciences, Washington, DC 20052, USA; acampbell@childrensnational.org

**Keywords:** sickle cell disease, soluble adhesion molecules, nitric oxide metabolites, ELISA

## Abstract

Nitric Oxide (NO) and soluble adhesion molecules are promising biomarkers, which predict endothelial dysfunction in sickle cell disease (SCD). Several studies have investigated the relationship between NO (as well as its metabolites) and endothelial adhesion molecules in SCD. However, these studies were done mainly in the developed world, and it is difficult to extrapolate the findings to SCD populations in other geographical regions such as Africa due to significant disparities in the results. The aim of the current study was to determine the correlation between levels of nitric oxide metabolites (NOx) and adhesion molecules in SCD patients in a tertiary hospital in Ghana. A case control cross-sectional study involving 100 SCD (made up of HbSS and HbSC patients) and 60 healthy controls was conducted. Concentrations of NOx and soluble endothelial adhesion molecules (ICAM-1, VCAM-1 and E-selectin) were measured in all the study participants (n = 160) by the Griess reagent system and enzyme-linked immunosorbent assay (ELISA). Correlation analysis was performed to determine a possible link between the variables. Levels of soluble adhesion molecules were higher in the HbSS patients. Correlation of NOx with ICAM-1 almost approached significance (r = 0.565, *p* = 0.058) in the HbSS patients. There were no correlations between NOx and E-selectin in both HbSS and HbSC patients. There were no significant correlations between NOx and VCAM-1 in all the study participants (*p* > 0.05). Of the soluble adhesion molecules, ICAM-1 showed a significant positive correlation with VCAM-1 in the HbSC patients. There were no significant differences between the adhesion molecules and the age of participants in the various study groups. Whether or not a significant correlation exists between NOx and soluble adhesion molecules may not depend on the sickle cell genotype. The expression of adhesion molecules may not depend on age.

## 1. Introduction

Sickle cell disease (SCD) is a group of inherited disorders characterized by an abnormal structure of one of the globin chains of the haemoglobin molecules, resulting in several clinical presentations [1]. The disease is known to be caused by a point mutation in the haemoglobin beta gene, which encodes the beta-globin found on chromosome 11a. In the globulin protein, the amino acid valine is substituted for glutamic acid as a result of the mutation. This leads to the synthesis of an abnormal form of the haemoglobin molecule, which is the haemoglobin S (HbS) [2]. Hyper-haemolysis is usually experienced by SCD patients, which possibly leads to a number of acute complications of the disease including vaso-occlusion, priapism, leg ulcers, and acute chest syndrome [3]. Among the several types of SCD, HbSS (often described as sickle cell anaemia), and haemoglobin SC disease (HbSC) are common [4]. In Ghana, there is an increase in the number of persons born with this disease condition such that, annually about 2% of neonates have SCD [5]. In the pathophysiology of SCD, soluble adhesion molecules (ICAM-1, VCAM-1, and E-selectin) often play a significant role in recruiting, as well as binding inflammatory cells to vascular endothelium [6]. Levels of these endothelial adhesion molecules have been reported to increase in patients with SCD [7,8,9], as well as other complications including acute chest syndrome [10].

Nitric oxide (NO) is a potent vasodilator produced by endothelial cells [11,12], and contributes to smooth muscle relaxation [13]. Nitric oxide (NO) plays a significant role in the pathogenesis of SCD as it is usually associated with the regulation of blood vessel tone and endothelial adhesion. In SCD, the levels of NO are brought down by free haemoglobin produced from haemolysis, leading to the production of more endothelial adhesion molecules [14]. Thus, chronic haemolytic episodes in severe clinical complications of SCD may result in the production of more endothelial adhesion molecules. Consequently, the levels of NO in these same patients may be down regulated, resulting in a possible endothelial dysfunction. The levels of NO as well as its metabolites are depressed in SCD and associated complications. 

In the clinical manifestations of SCD, levels of NO metabolites are particularly reduced during a vaso-occlusive crisis [15,16,17]. NO is involved in the suppression of adhesion molecule expression on endothelial cells [18]. Several studies have investigated the relationship between NO (as well as its metabolites) and endothelial adhesion molecules in SCD [19,20,21,22,23,24]. However, these studies were done mainly in the developed world, and it is difficult to extrapolate the findings to SCD populations in other geographical regions such as Africa due to significant disparities in the results. In Ghana and several other sub-Saharan African countries, little is known about the relationship between NO (as well as its metabolites) and endothelial adhesion molecules in SCD. Given that these parameters are promising biomarkers and predict endothelial dysfunction in SCD, it is worthwhile understanding their relationship, especially in sub-Saharan Africa where the majority of SCD cases occur. The current study aimed to determine a possible correlation between NO metabolites and soluble adhesion molecules in Ghanaian SCD patients.

## 2. Methods

### 2.1. Study Site

The study was conducted at the Korle-Bu Teaching Hospital (KBTH) in the Greater Accra Region of Ghana. The hospital is a tertiary one with a bed capacity of about 2000, where several patients are referred for health care. The average daily attendance of patients in this hospital is 1500. 

The sickle cell unit of KBTH wherein the current study was undertaken, is known to have 25,000 registered SCD patients and a daily attendance of 40.

### 2.2. Study Design, Subject Recruitment, and Data Collection

The study was a cross-sectional one involving 100 SCD patients (HbSS and HbSC) and 60 controls, conducted at KBTH from June to September 2015. Patient recruitment was based on the clinical diagnosis of SCD. SCD patients with diabetes mellitus, hypertension, or coronary artery disease, as well as those who have received blood three months prior to the study were excluded. Recruitment of cases was done at the sickle cell unit where patients come for their routine hospital attendance. Patients’ genotypes were determined using haemoglobin electrophoresis. The controls were recruited from the National Blood Transfusion Centre located at KBTH. They were apparently healthy people and were aged- and sex-matched to the SCD patients. Information on demographic features of the study participants as well as clinical data obtained from patients’ medical history were gathered.

### 2.3. Laboratory Analysis

Venous blood samples (5 mL) were obtained from each of the study participants for nitric oxide metabolite (NOx) and soluble adhesion molecule assays. Blood samples were drawn from the antecubital region of the arm. The tourniquet was not left on the arm for more than one minute. The venipuncture site was allowed to completely air dry after cleaning with alcohol. The needle was placed correctly in the vein, while the plunger of the syringe was pulled gently. Blood samples were collected from both subjects (controls and SCD patients) into ethylendiaminetetracetic acid (EDTA) tubes, and mixed gently to minimize haemolysis. They were centrifuged, separated and plasmas obtained were stored at –80 °C. An aliquot of the plasma was used to determine nitrite and adhesion molecules for both SCD patients and the control group. The Griess assay, which is based on the chemical reaction of sulfanilamide and N-l-naphthyl ethylenediamine dihydrochloride (NED) under acidic (phosphoric acid) conditions, was used to measure nitrite. Triplicates of each experimental sample were done and the average absorbance was recorded. The Nitrite Standard Reference Curve was used as a reference point with which nitrite concentration (Y) of each experimental sample was compared with. The standard curve-generated formula Y = 0.0185X + 0.106 was used; where X is the average absorbance of the experimental sample.

To determine plasma levels of VCAM-1, ICAM-1, E-Selectin concentrations, human VCAM-1, ICAM-1, and E-Selectin, ELISA kits (GenWay, San Diego, CA, USA, ELISA Development kit) were used, according to the manufacturer’s protocol. The plasma concentrations of VCAM-1, ICAM-1, and E-Selectin were measured in duplicate. The method described by Antwi-Boasiako et al. [9] was used to quantify these soluble adhesion molecules. The optical density absorbance for the adhesion molecules was read at 450 nm in a microplate reader (Amersham Bioscience Limited, Buckinghamshire, UK).

### 2.4. Data Analysis

Data from the study was entered in Microsoft Excel 2010, and analyzed in SPSS version 20 software (IBM Corporation, Armonk, NY, USA). The results were expressed as mean plus or minus standard deviation (mean ± SD). The Kruskal Wallis test was used to compare differences in mean values among SCD patients and the control group. Correlation analysis was run to determine a possible association between NOx and the soluble adhesion molecules, as well as the ages of the study participants. Statistical significance was considered at *p* < 0.05.

### 2.5. Ethics Statement

Ethical approval for the study was sought from the Ethical and Protocol Review Committee of the College of Health Sciences, University of Ghana Medical School (Protocol identification number + MS-Et/M.11–P 5.7/2012–2013). Study participants provided their blood samples as well as demographic information after the study procedure was thoroughly explained to them, and following their consent to partake in the study.

## 3. Results

### 3.1. Demographic and Clinical Characteristics of the Study Participants

A total of 160 participants were recruited into the study, consisting of 100 SCD patients and 60 controls. Of the SCD patients, 80 were HbSS and 20 were HbSC genotype. The mean age of the study participants were 25.9 ± 8.6 for HbSS patients, 34.1 ± 12.2 for HbSC patients and 32.3 ± 10.24 for the controls.

### 3.2. Plasma Concentrations of Circulating Adhesion Molecules, Nitric Oxide, and Haemoglobin Levels in the Study Participants

As expected, levels of circulating adhesion molecules were higher in the HbSS and HbSC patients compared to the control group. Nitric Oxide metabolite (NOx) levels as well as Hb levels were however, decreased in the sickle cell patients (Table 1).

### 3.3. Correlation between Plasma Nitrite and Adhesion Molecules

Levels of NOx showed positive insignificant correlation with E-selectin in the control group (Table 2). In both HbSS and HbSC individuals, there was no correlation between NOx and E-selectin. Nitric Oxide metabolites correlated positively (and weakly) with ICAM-1 and VCAM-1 in HbSS, although there were no significant differences amongst them. Compared with the HbSS patients, there was a negative weak correlation of NOx with ICAM-1 in HbSC patients (Table 3 and Table 4).

Whereas no correlation of ICAM-1 and VCAM-1 was realized in HbSS patients, there was a strong negative association in HbSC patients (r = −0.002, *p* = 0.985 versus r = −0.528, *p* = 0.017). No correlation existed between ICAM-1 and E-selectin in HbSS patients compared to a weak negative correlation of ICAM-1 and E-selectin in HbSC patients (r = 0.096, *p* = 0.335 versus r = −0.219, *p* = 0.383). VCAM-1 correlated positively with E-selectin in HbSC patients, but no correlation was observed in HbSS patients (r = 0.144, *p* = 0.569 versus r =−0.077, *p* = 0.444) (Table 3 and Table 4).

There were no significant correlations between the soluble adhesion molecules and age in the study populations. There were positive correlations only between the adhesion molecules and the age of healthy individuals (Controls) as well as SCD patients with the HbSC genotype in the current study (Table 5).

## 4. Discussion

In this study, we investigated the correlation between NOx and soluble adhesion molecules (ICAM-1, VCAM-1, and E-selectin) in Ghanaian SCD patients and observed that, NOx correlated insignificantly with some of the adhesion molecules in the study participants. Associations between NOx and endothelial adhesion molecules have been demonstrated in other disease conditions elsewhere [24]. A recent study on chronic obstructive pulmonary disease revealed that, NOx levels correlate insignificantly with ICAM-1 [24]. Nitrite levels correlated with levels of VCAM-1 in patients with systemic lupus erythematous in a previous study [23]. Findings from this study, however, suggest in part that there may be no significant correlations (if any) between NOx and adhesion molecules in sickle cell disease patients with either HbSS genotype or HbSC genotype in Ghana.

Although previous studies have reported significant correlations between some soluble adhesion molecules, particularly the expression levels of VCAM-1 and age in several disease complications [19,20,21], the current study reported insignificant correlations between the parameters. In the same study conducted by Richter et al. [21] among patients with vascular diseases, circulating E-selectin levels did not correlate with age. Results from this present study suggest in part that, expression levels of adhesion molecules in SCD may not be age dependent. The differences could be partly attributed to the age range of the study participants recruited, among the various studies. The intensity of haemolysis and the underlying chronic condition in SCD patients may, in part, play a role in the interaction between NO and its metabolites, and endothelial adhesion molecules. In principle, there are several acute complications associated with SCD, where a possible significant association between NO or perhaps its metabolites and endothelial adhesion molecules may be appreciated [14]. It is worth mentioning that, the current study measured NO metabolites and not NO. This could partly explain the observed insignificant correlation between NOx and the adhesion molecules in the study populations. 

Of the adhesion molecules studied, VCAM-1 correlated positively and insignificantly with NOx in SCD patients. Observations from the current study suggest that an elevation of NOx may cause a corresponding rise in VCAM-1 in SCD patients. Several other studies have reported that levels of VCAM-1 are particularly increased in severe complications of SCD, including HbSS vaso-occlusive crises [7,8,9] and acute chest syndrome [10], and thus, may serve as a promising biomarker of disease severity and associated complications of SCD [9]. Although levels of NO as well as its metabolites and soluble adhesion molecules have been exploited in single cohort studies among SCD patients [7,8,9,17,25], a correlation between the two has not been given a closer look. In another study, a significant correlation was observed between nitrate and endothelial adhesion molecules in patients with systemic sclerosis [26]. The relative levels of NO and soluble endothelial adhesion molecules are very important in the pathophysiology of SCD. Indeed, higher levels of adhesion molecules suppress the expression of NO, and this is mostly encountered in SCD patients and even transgenic mice [8,27]. 

The current study indicates that, apart from healthy individuals, there was no correlation between NOx and E-selectin in HbSS and HbSC patients, although they are different genotypes. This may partly suggest that there were similar degrees of haemolysis in these two separate patients. Thus, a similar ongoing vasculopathy experienced by these patients in our study may, in part, explain this observation. Results from the current study suggest, in part, that significant correlation of NOx with endothelial adhesion molecules may not depend on the actual sickle cell genotype. Nonetheless, we cannot overcome the fact that levels of endothelial adhesion molecules were elevated in patients with the HbSS genotype in the present study. We have previously reported a continuous rise in ICAM-1, VCAM-1, and E-selectin in HbSS patients with sub-phenotypes including priapism and leg ulcers at a tertiary hospital [9]. The study indicated that, levels of adhesion molecules increased remarkably in HbSS patients with vaso-occlusive crises compared to those in the steady state with the HbSS genotype [9]. Although NO donors have been implicated to reduce expression of endothelial adhesion molecules, it appears that, different NO donors may in part, regulate the expression of adhesion molecules differently (whether significant association or insignificant association).

## 5. Conclusions

The current study reports that, although the levels of adhesion molecules were higher in HbSS patients, they correlated insignificantly with NOx. While there were no significant associations between NOx and the endothelial adhesion molecules in controls and patients with HbSS and HbSC genotype, ICAM-1 correlated negatively and significantly with VCAM-1 in the HbSC patients. Thus, HbSC patients in our population will have higher ICAM-1 and lower VCAM-1 levels, and vice versa. Future studies could focus on a possible association between NO as well as arginine and adhesion molecules in SCD patients with acute complications. Association of NO and arginine with markers of haemolysis could also be investigated among people with African ancestry.

## Figures and Tables

**Table 1:** Plasma concentrations of circulating adhesion molecules, Nitric Oxide metabolites, and haemoglobin levels in the study participants.

Parameter	ICAM-1 (ng/mL)	VCAM-1 (ng/mL)	E-Selectin (ng/mL)	NOx (µMol/L)	Hb (g/dL)
HbAA	29.60 (12.03–40.32)	286.10 (179.36–356.21)	157.49 (138.96–543.53)	59.66 ± 0.75	14.30 ± 3.50
HbSC	31.67 (13.56–68.94)	540.32 (258.73–876.37)	219.44 (92.51–829.49)	47.73 ± 11.14	11.63 ± 1.53
HbSS	54.16 (25.67–77.20)	502.85 (289.12–876.77)	221.68 (99.79–842.65)	30.87 ± 1.25	8.88 ± 1.83
*p*-value	<0.05	<0.05	<0.05	<0.05	<0.05

*p* value <0.05 is significant. Hb—haemoglobin; HbAA—haemoglobin normal; HbSC—haemoglobin sickle cell disease; HbSS— sickle cell anaemia.

**Table 2:** Correlation between levels of nitric oxide metabolites (NOx) and the levels of soluble adhesion molecules in controls.

Parameters	ICAM-1	VCAM-1	E-selectin
ICAM-1	Nil	r = −0.156, *p* = 0.371	r = 0.248, *p* = 0.172
VCAM-1	r = −0.156, *p* = 0.371	Nil	r = 0.044, *p* = 0.81
E-selectin	r = 0.248, *p* = 0.172	r = 0.044, *p=* −0.81	Nil
NOx	r = −0.252, *p* = 0.164	r = 0.047, *p* = 0.797	r = 0.059, *p* = 0.748

r = Pearson correlation coefficient; *p* value <0.05 is significant.

**Table 3:** Correlation between levels of NOx and levels of soluble adhesion molecules in HbSS patients.

Parameters	ICAM-1	VCAM-1	E-Selectin
ICAM-1	Nil	r = −0.002, *p* = 0.985	r = 0.096, *p* = 0.335
VCAM-1	r = −0.002, *p* = 0.985	Nil	r = −0.077, *p* = 0.444
E-selectin	r = 0.096, *p* = 0.335	r = −0.077, *p* = 0.444	Nil
NOx	r = 0.565, *p* = 0.058	r = 0.121, *p* = 0.230	r = −0.020, *p* = 0.840

r = Pearson correlation coefficient; *p* value < 0.05 is significant.

**Table 4:** Correlation between levels of NOx and levels of soluble adhesion molecules in HbSC patients.

Parameters	ICAM-1	VCAM-1	E-Selectin
ICAM-1	Nil	r = −0.528, *p* = 0.017	r = 0.219, *p* = 0.383
VCAM-1	r = −0.528, *p*= 0.017	Nil	r = 0.144, *p* = 0.569
E-selectin	r = −0.219, *p*= 0.383	r = 0.144, *p* = 0.569	Nil
NOx	r = −0.080, *p*= 0.746	r = 0.161, *p* = 0.511	r = −0.014, *p* = 0.957

r = Pearson correlation coefficient; *p* value <0.05 is significant.

**Table 5:** Correlation between levels of soluble adhesion molecules and age among the study participants.

Parameters	Age
ICAM-1 (Controls)	r = 0.043, *p* = 0.807
VCAM-1 (Controls)	r = 0.115, *p* = 0.511
E-selectin (Controls)	r = 0.079, *p* = 0.667
ICAM-1 (HbSC)	r = 0.318, *p* = 0.199
VCAM-1 (HbSC) E-selectin (HbSC)	r = 0.113, *p* = 0.656 r = 0.074, *p* = 0.770
ICAM-1 (HbSS)	r = −0.062, *p* = 0.606
VCAM-1 (HbSS)	r = 0.086, *p* = 0.393
E-selectin (HbSS)	r = −0.056, *p* = 0.577

r = Pearson correlation coefficient; *p* value <0.05 is significant.
